# Tuning of the band gap and dielectric loss factor by Mn doping of Zn_1-x_Mn_x_O nanoparticles

**DOI:** 10.1038/s41598-023-35456-2

**Published:** 2023-05-27

**Authors:** Wiqar Hussain Shah, Azeema Alam, Hafsa Javed, Khadija Rashid, Akhtar Ali, Liaqat Ali, Akif Safeen, Muhammad R. Ali, Naveed Imran, Muhammad Sohail, Gilbert Chambashi

**Affiliations:** 1 Department of Physics, Faculty of Sciences, International Islamic University, H-10, Islamabad, Pakistan; 2 Department of Physics, University of Poonch Rawalakot, Rawalakot, 12350 Pakistan; 3 Faculty of Engineering and Technology, Future University in Egypt, New Cairo, 11835 Egypt; 4 Department of Applied Mathematics and Statistics, Institute of Space Technology, 2750, Islamabad, 44000 Pakistan; 5 HITEC Colleges, HIT Taxila Cantt, Taxila, Pakistan; 6 Institute of Mathematics, Khwaja Fareed University of Engineering & Information Technology, Rahim Yar Khan, 64200 Pakistan; 7 School of Business Studies, Unicaf University, Longacres, Lusaka, Zambia

**Keywords:** Materials science, Nanoscience and technology

## Abstract

This study explored the structural, optical, and dielectric properties of Pure and Mn^+2^ doped ZnO nano-particles (Zn_1−x_Mn_x_O) with x ≥ 20%, synthesized by co-precipitation method followed by annealing at 450^0^C. Different characterization techniques were conducted to characterize the as-prepared nano-particles. X-ray Diffraction analysis of the pure and Mn^+2^ doped presented a hexagonal wurtzite structure and a decreased crystallite size with increasing doping concentration. Morphological analysis from SEM revealed finely dispersed spherical nanoparticles with particle size of 40–50 nm. Compositional analysis from EDX confirmed the incorporation of Mn^+2^ions in ZnO structure. The Results of UV spectroscopy showed that changing the doping concentration affects the band gap, and a red shift is observed as the doping concentration is increased. The band gap changes from 3.3 to 2.75 eV. Dielectric measurements exhibited decrease in the relative permittivity, dielectric loss factor and ac conductivity by increasing Mn concentration.

## Introduction

Among metal oxides, Zinc oxide has always been important for researchers as it has been employed in the past in various ceramics and pharmaceuticals^1^. Recently, it’s again in the spotlight because of modifications in the physical properties of material have found remarkable applications^2^. In the field of optoelectronics and photonics, manipulating the band gap is the key stone for many practical devices^3^. Due to the diverse properties ZnO possess, it’s reported in literature in fabricating piezoelectric transducers, optical waveguides^4^, transparent conductive oxides, chemical and gas sensors^5^, spin functional devices, and UV-light emitters^6^. Zinc Oxide with a wide band gap of 3.37 eV, when doped has great potential for a variety of application including photo sensors, photodiodes, lasers, solar cells and LED’s at room temperature as compared to GaAs^7^. ZnO nanowires have been reported in solar cells to enhance the efficiency^4^. Transition metal doped ZnO with a direct band gap in the visible range makes it attractive as photosensitive and a light absorbing material^8^.V.D. Mote et al. reported that Mn doped ZnO has gained certain interest again because of doping which give it its dilute magnetic semiconducting nature and make it useful for spintronics. Low energy consumption and high efficiency can be achieved at room temperature owing to the large exciton binding energy of 60 meV^8^. Zinc Oxide is employed as a buffer layer, transparent conducting oxide and as an intermediate layer in various devices so, tailoring the band gap with doping paves way for metal oxide based photovoltaic which are cost effective as compared to silicon based devices. Multi junction solar cells can be created with different amount of doping so to absorb maximum range of wavelengths in visible light. ZnO has been reported being used in combination with TiO_2_, with ZnO having better conductivity and TiO_2_contributing to decrease the rate of recombination owing to its fewer defect states^9^.

Shakeel khan et al. reported on the dielectric properties of Mn-doped ZnO; these properties change as the temperature and type of material changes, so as the doping concentration changes so does these properties. The results encourage the use of Mn-doped ZnO in devices operating at high frequencies^10^. Dinesha et al. also reported on the structural and dielectric behavior of Fe doped ZnO and attributed the increase in ac conductivity on the basis on increasing hopping mechanism^11^. They suggest that the study on the dielectric behavior of Mn-doped ZnO is very useful. In modern day semiconductor technology speeding electron transportation and reducing losses is important. DSSCs or dye-sensitized solar cells are a class of excitonic photocells that are efficient and very stable for energy generation^12^. The basic idea is to combine ZnO nanoparticles and ZnO nanowires to make a photoanode which provides a large surface area for absorption as well as enhances electron transport which can be further improved by Mn doping^13^. Fabbiyola et al*.* reported that the comparison between the ionic radii of Mn^+2^ and Zn^+2^ reveals they are quite similar and thus produce a good, high solubility Mn-doped ZnO crystal structure as compared to other transition metals^14^.

It has been reported that the ZnO band gap might be decreased by adding a suitable transition metal to the crystal lattice of ZnO in order to provide new energy levels immediately below the conduction band^15^. When the coordination environment of Zn in the ZnO structure is altered by the foreign elements inserted, ZnO's electronic structure is altered, which improves its photo-catalytic effectiveness. In a recently published study on cobalt-doped titania, this phenomenon was emphasized. Notably, s-d and p-d interactions significantly impact the d^n^ electronic configuration of transition metals when they are utilized as foreign doping elements. The quantity of the doping element utilized, on the other hand, has been shown to have an impact on the doped oxide's structural, optical, and photocatalytic characteristics, as has been mentioned in several research^16–19^.

Modern-day technology emphasizes controlling and manipulating properties of materials, so in this investigation, we report on tuning the band gap as well as the dielectric properties simultaneously with Manganese doping up to 20% without affecting the structure of Zinc Oxide. Several methods have been reported on the fabrication of Mn-doped ZnO, including sol–gel route^9^, RF Magnetron sputtering^12^, hydrothermal method^13^, and co-precipitation^6^. We have employed co-precipitation, as this process can avoid complex steps at less temperatures thus resulting in less time consumption than other techniques. Different characterizations have been used to study the prepared samples, including x-ray diffraction, scanning electron microscopy, energy dispersive x-ray spectroscopy, UV–vis spectroscopy, and dielectric analysis.

## Experimental procedure

The samples of Mn substituted ZnO with nominal compositions of Zn_1-x_Mn_x_O $(\mathrm{x}=0\mathrm{\%},5\mathrm{\%}, 10\mathrm{\%},15\mathrm{\%},20\mathrm{\%})$ were synthesized by co-precipitation techniques. In this technique, aqueous salt solutions of reactants have been mixed to produce precipitation of insoluble substances by exceeding the solubility limit Zinc acetate dehydrates Zn(CH_3_COOH)_2_.2H_2_O, Manganese acetate tetrahydrate Mn(CH_3_COOH)_2_.4H_2_O, and sodium hydroxide NaOH of analytic grade were used in this experiment without further purification. To prepare the un-doped ZnO sample, the appropriate amount of Zinc acetate di-hydrate Zn(CH_3_COOH)_2_.2H_2_O was dissolved in 300 ml of distilled water along with adding NaOH drop by drop for adjusting the pH up to 8.5.The average expected size at this pH is 40 nm. The solution was stirred on a hot plate for two and half hours using a magnetic stirrer. Temperature was maintained at 80–85 °C. The size of nanoparticles has been controlled by optimizing different synthesis parameters such as pH value, concentration of dopants, and reaction time. The solution was then cooled to room temperature. The precipitates formed were washed several times with distilled water. Then these samples were dried in an oven at 150 °C for 1 h and 30 min. The prepared samples were annealed at 450 °C in a furnace for 4 h to improve their physical properties. For Mn-doped ZnO, we added Manganese acetate tetrahydrate Mn(CH_3_COOH)_2_.4H_2_O and Zinc acetate dehydrate Zn(CH_3_COOH)_2_.2H_2_O in a Stoichiometric ratio in 300 ml of distilled water and the same procedure was followed as for doped sample for 0.05, 0.10, 0.15, and 0.20.

X-ray Diffraction has been used to examine the structure using Cu-kα radiations as source with a wavelength of 1.54 Å, with 2θ in the range from 20° to 70°. Morphological analysis was done using a Scanning electron microscope (VEGA TESCAN-13 AT NUST) at 20 keV. Dielectric parameters were investigated using an Agilent E4980 LCR meter in the frequency range (20 Hz to 2 MHz), and UV–vis spectroscopy gave us the energy band gap, with absorption spectra in the range from 200 to 1000 nm.

## Results and discussion

**Figure 1:**
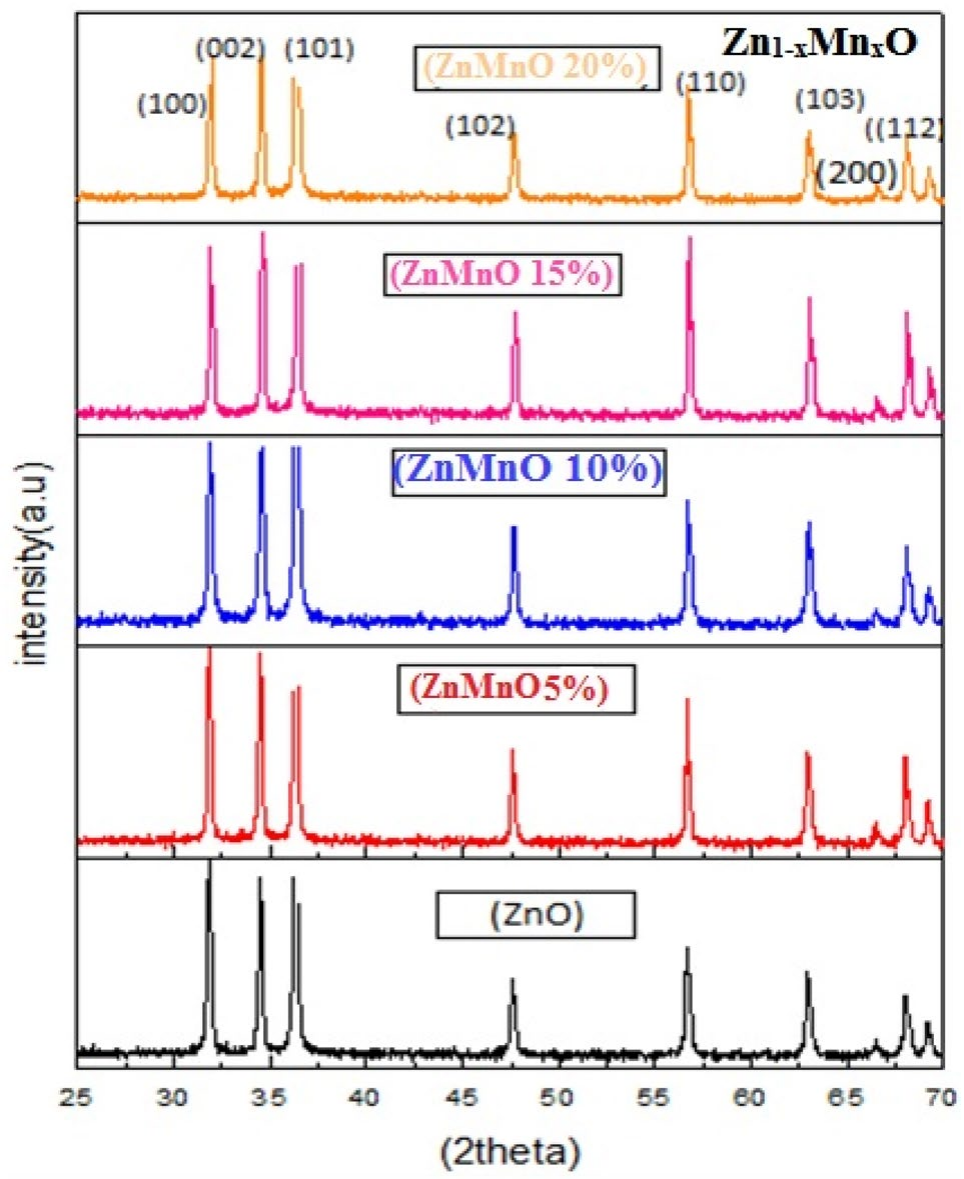
XRD pattern between 2θ and intensity for pure and Mn doped ZnO.

X-ray diffraction (Diffractometer system = XPERT-3 Malvern Panalytical) by using Cu Kα radiation having wavelength 1.54 Ao over the angular range of 20° ≤ 2θ ≤ 70° by step scanning results at a step size of 0.02° at counting time of 3 s per step). It is a well-known technique through which we can analyze the structural properties of a material. Figure 1 shows the XRD data for un-doped and Mn doped ZnO at different doping concentrations (0.00, 0.05, 0.10, 0.15, and 0.20). The sharp peaks represent a good level of crystallinity in these samples; also, secondary phases are observed, suggesting pure and single-phase samples. The diffractogram showed three broad peaks for un-doped and Mn-doped ZnO, these peaks match well to the crystalline plane of a hexagonal wurtzite structure. The peaks for doped and un-doped ZnO nanoparticles are similar, which shows that Mn^+2^ ions have successfully replaced Zn^+2^ions for up to 20% of doping. The crystallite size for all samples is calculated using the Debye sheerer formula^14^. The Table1 shows a decreasing trend in crystallite size with increasing doping concentration, from 40 nm pure ZnO to 50 nm for 20% Mn-doped ZnO. Peak broadening can be observed with increasing doping concentration, which affects the particle size. The cause of this change is the lattice strain produced in the system due to slightly greater radii of Mn^+2^ion than Zn^+2^ ions^18^, whereas the dislocation density can be calculated by Eq. (1)^22^.

1 $$\delta \, = {1}/{\text{ D}}^{{2}}$$

**Table 1:** Values of crystallite size for un-doped and Mn doped ZnO nanoparticles.

Doping concentration	Crystallite size	Lattice constant(a)nm	Lattice constant(c)nm	a/c	Unit cell volume	Dislocation density(1/m^2^)
0.00	36.90	0.324	0.529	0.562	0.04	7.344 × 10^14^
0.05	34.11	0.323	0.560	0.576	0.052	8.594 × 10^14^
0.10	28.91	0.311	0.540	0.575	0.048	1.196 × 10^15^
0.15	27.51	0.298	0.517	0.576	0.037	1.321 × 10^15^
0.20	23.06	0.299	0.598	0.50	0.046	1.880 × 10^15^

The variation in the crystallite size, lattice constants, volume, and dislocation density with doping concentration are shown in the Table1. The crystallite size decreases unit cell volume by doping Mn in ZnO. This may be due to the lattice strain produced in the system due to greater radii of Mn^+2^ion than Zn^+2^. The addition of Mn to ZnO has significant effect on the dislocation density of the resulting material. The dislocation density decreases. The decrease in dislocation represents a decrease in the content of lattice imperfections and indicates the formation of better quality samples^20,21^.

**Figure 2:**
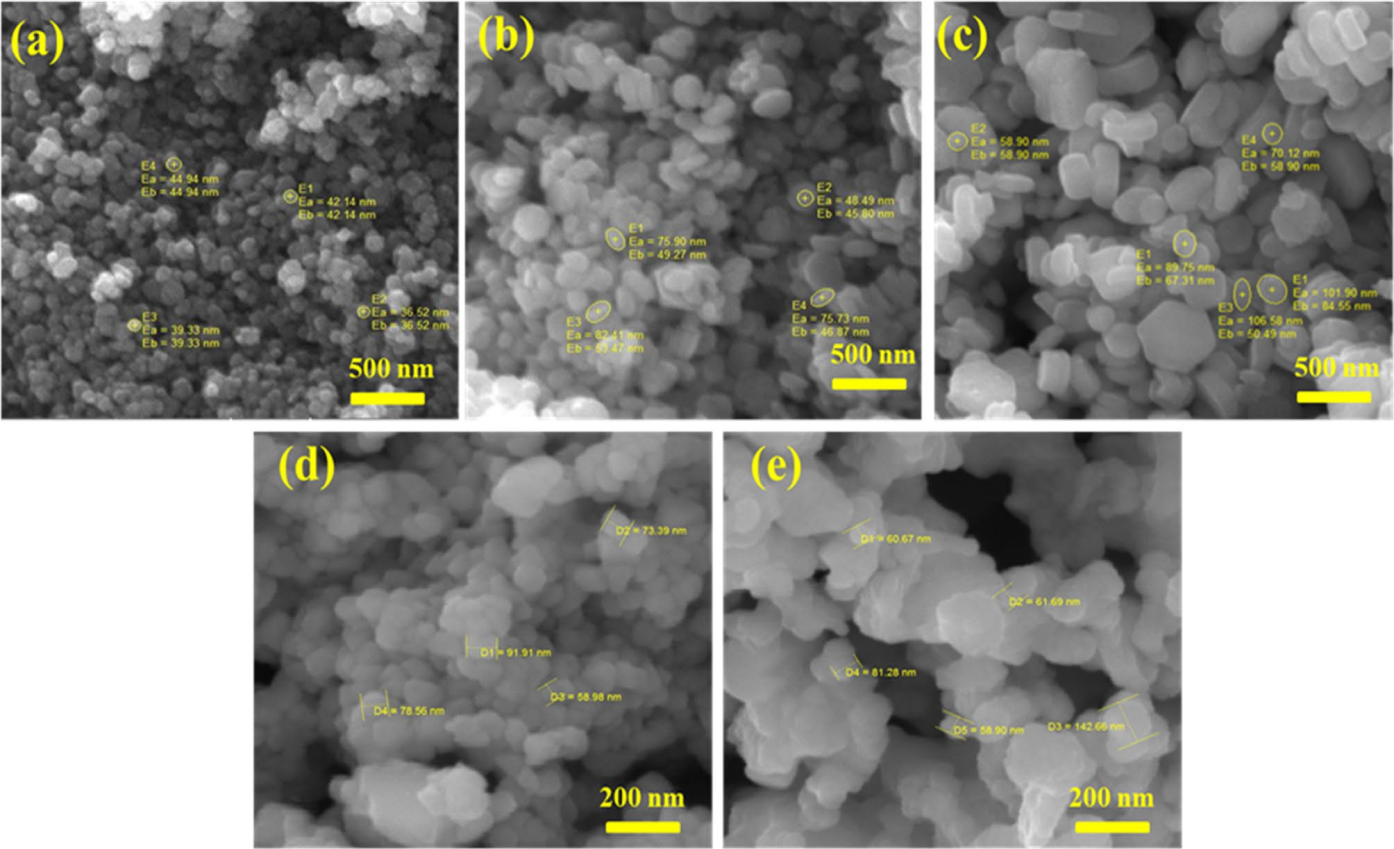
(**a**) SEM image for PURE ZnO (**b**) SEM image for 5% doping (**c**) SEM image for 10% (**d**) SEM image for 15% (**e**)SEM image for 20% Mn-based ZnO nanoparticles.

Morphological analysis of these samples has been investigated using scanning electron microscopy (SEM).SEM images are shown in the Fig. 2a for un-doped ZnO and(b),(c), (d),(e) for 5%, 10%, 15% and 20% Mn doped ZnO respectively. The SEM images revealed that the particles are spherical with a little bit of alteration as the doping concentration increased. The particle size observed ranges from 40 to 50 nm. The SEM images show a spherical morphology and agglomeration is observed as doping concentration increases.

**Figure 3:**
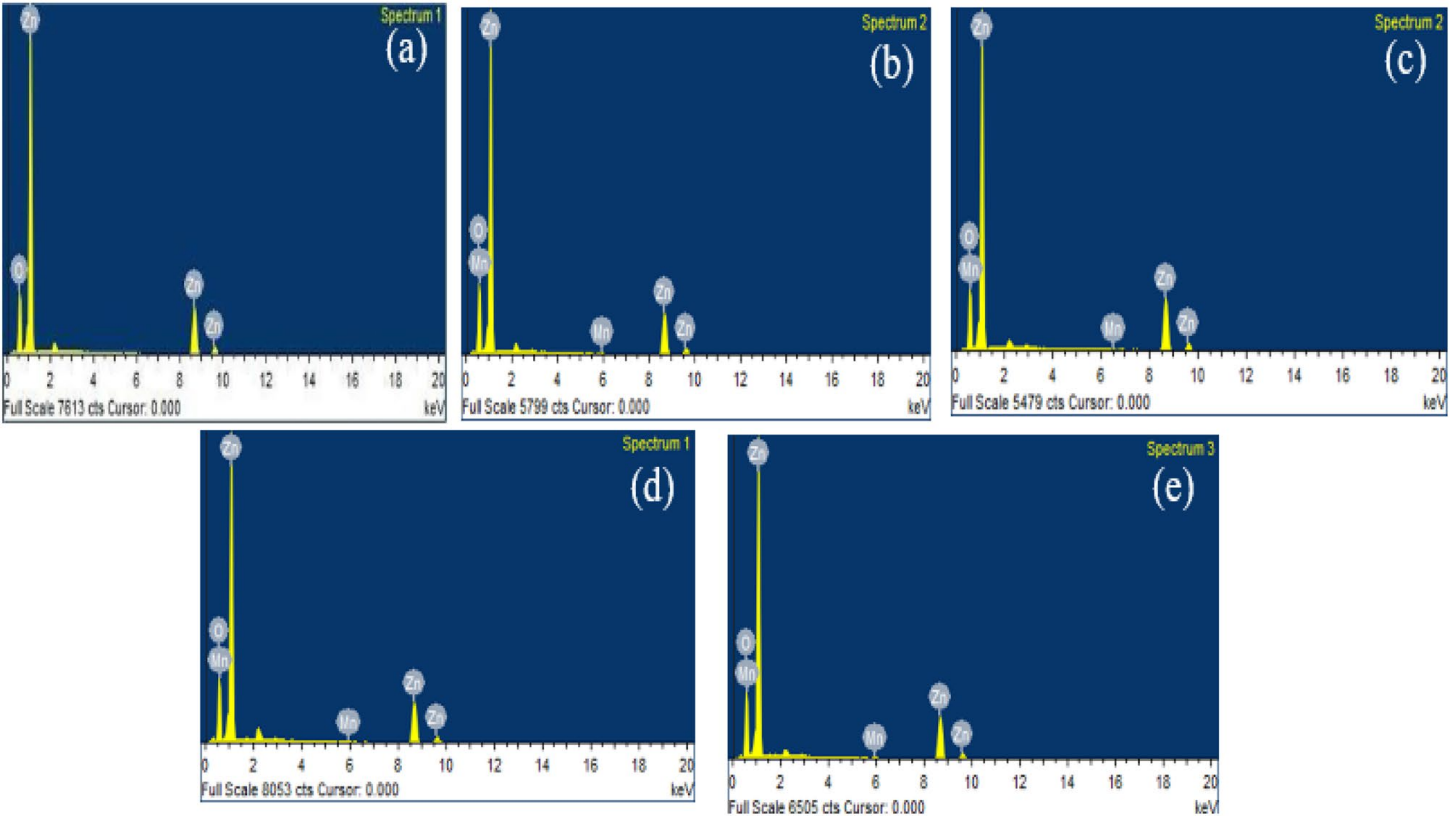
EDX analysis (**a**) un-doped ZnO energy spectrum (**b**) 5% Mn-doped ZnO energy spectrum (**c**) 10% doped ZnO energy spectrum (**d**) 15% doped ZnO energy (**e**) 20% doped spectrum of ZnO nanoparticles.

The elemental analysis was done using an EDS (Energy dispersive spectroscope).Fig. 3a shows EDX for un-doped ZnO, which reveals no traces of unwanted elements, confirming the purity of ZnO. On the other hand, Figure 3b–e shows 5%, 10%, 15%, and 20%Mn doped ZnO, and it shows the presence of Manganese in addition to Zn and O. The initial and observed weight percentages match and the atomic and weight percentage corresponding to different doping are shown in Table 2.

**Table 2:** Quantitative analysis from energy dispersive spectroscopy for un-doped ZnO and 5%, 10%, 15%, and 20% Mn-doped ZnO.

Sample	Zn (at %)	O (at)%	Mn (at) %
ZnO	27.89	72.41	0
ZnMnO 5%	25.27	74.68	0.05
ZnMnO 10%	30.85	69.09	0.09
ZnMnO 15%	24.15	75.70	0.15
ZnMnO 20%	22.67	77.12	0.20

**Figure 4:**
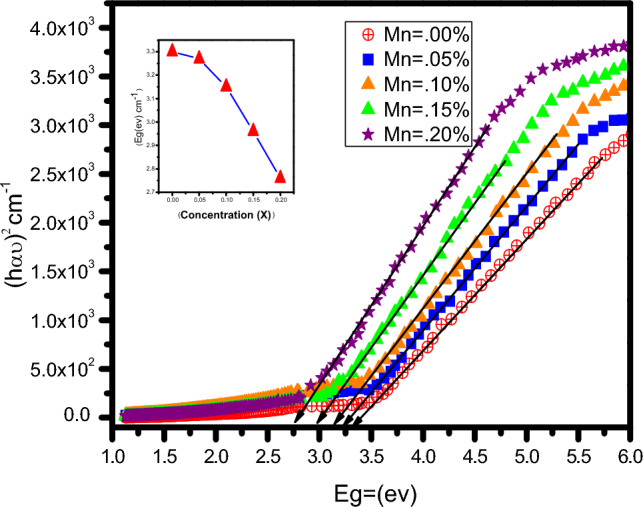
Tac plots of the prepared nanostructures (the Inset of the figure shows the band gap energy calculation).

At room temperature, UV visible spectroscopy (SPECORD 200 PLUS) was conducted on pure and Mn doped ZnO. The maximum absorption range of wavelength was noted for a pure sample. It was passed through other doped samples, which provided us with the wavelength and the relative optical transmittance of the sample. Further calculations were carried out from these quantities. The maximum absorption range was found to be between 200 and to1000 nm. Before doping, pure sample was found to be transparent in the visible spectrum, only showing absorption peaks in UV region. As the doping concentration is raised up to 20%, the spectra correspond to peaks in the visible region with a wavelength corresponding to 427 nm. The variation in the band gap is studied using the Tauc relation as given in Eq. (2).

2 $$(\alpha {\text{hv}}) ^{{2}} = {\text{A}}({\text{hv}}{-}{\text{E}}_{{\text{g}}} ) ^{{\text{n}}}$$

where n is ½ for direct band gap materials and 2 for indirect band gap materials, the plot between (αhν)^2^and hν is shown in Fig. 4. Extrapolating the curve's linear part to the x-axis provides the direct band gap for each sample. The inset graph shows relation between the band gap and doping concentration. It can be observed that with the increase in doping concentration of 5%, 10%, 15%, and 20%, the band gap changes from 3.25 eV, 3.12 eV, 3.0 eV, and 2.75 eV.This change in the band gap can be attributed to the s-p-d exchange interaction between the dopant Mn^+2^ and the host ZnO. The doping of Mn^+2^ in the ZnO lattice created impurity levels. And as a result, the d orbital of Mn overlaps with the 2p orbital of oxygen and the 4 s orbital of Zn. This causes an exchange interaction between these orbitals, raising the valence band maximum and lowering the conduction band minimum, respectively. This demonstrates that the band gap can be tuned by changing the dopant concentration. The tuning of the band gap is important characteristic that allows ZnO to be used in photovoltaic and thermoelectric. The band gap is also influenced by structural deformation. These deformations may result in piezoelectric polarization in the system, creating local electric fields which produce band-bending effects^14,18^.

**Figure 5:**
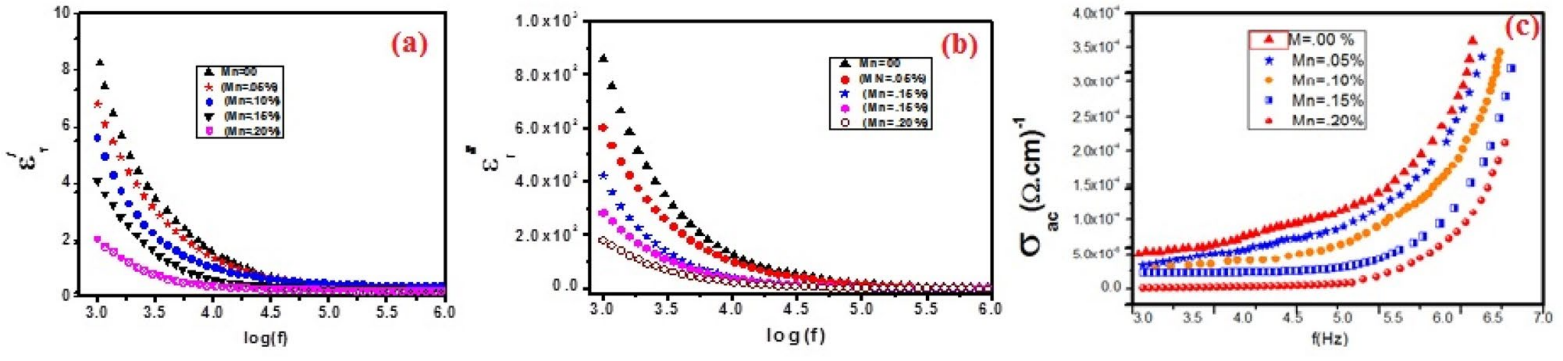
(**a**) Frequency-dependent dielectric constant, (**b**) dissipation factor or tangent loss and (**c**) ac conductivity of Mn-based ZnO nanoparticles.

The dielectric studies were carried out using an LCR meter. The sample in the form of a pellet was put in between a parallel plate capacitor. An AC supply is applied across the plates and the corresponding frequency and capacitance are measured to find the real and imaginary part of permittivity. The real part that is the dielectric constant is given by Eq. (3):

3 $$\varepsilon ^{\prime} = ({\text{t}} \times {\text{C}})/({\text{A}} \times \varepsilon_{0} )$$

where A is the area of the pellet, C is the capacitance, ε_0_ is the permittivity of free space, and t is the thickness of our pellet. The dielectric constant versus frequency for all samples is shown in Fig. 5. Dielectric properties of a material are dependent on frequency, and it can be noticed that the dielectric constant decreases with increasing frequency, among all polarizations taking place including atomic, ionic, dipole, and space charge polarization. Space charge polarization is most significant in the case of heterogeneous structures. This phenomenon can be explained on the basis of the Maxwell–Wagner Model. In heterogeneous materials, charge carriers tend to accumulate at the interface or boundaries. According to the model as mentioned above, in a dielectric material, two types of regions are formed on applying ac supply, namely grains and grain boundaries. Where grain is the conductive region in the dielectric and grain boundaries are the insulating walls between grains. So, at low frequencies, the grain boundaries play an active role, and a charge is build up at the grain boundaries, while at high frequencies, the dipole moment is notable to orient fast enough to keep in alignment with the applied field,so the dielectric constant becomes independent of the applied frequency. The doping effect also decreases the dielectric constant because as the doping concentration increases, more defects are introduced which tend to increase the grain boundary's thickness and thus reduce the amount of charge accumulated^9^. Figure 5a shows the plot between dielectric constant variations with doping concentrations.

The dielectric loss, also known as the loss factor, is the efficiency with which electromagnetic radiations are converted to heat. Mathematically it is written as given in Eq. (4).

4 $$\varepsilon^{^{\prime\prime}} = \varepsilon \;\tan \;\delta$$

where ε″ is the dielectric loss, tanδ is the dielectric loss tangent. The graph between dielectric loss dependent on frequency is shown in Fig. 5b. The behavior shown in the plot can be explained on basis of Koop’s Model which is like Maxwell Wager Model. According to this theory, at low frequency the time between polarization and depolarization is large and the loss is greater while as we go towards higher frequencies the dipole moment does not respond to the electric field and becomes independent of it. Fundamentally, to orient dipoles in the direction of electric field energy is dissipated due to the resistance from their inertial mass. At low frequencies, the polarization lags the applied field thus producing a greater loss factor. ZnO is a polar molecule, but as we introduce Mn + 2 it reduces the polarity of the doped sample, thus decreasing the dielectric constant and the loss factor.

The variation in conductivity with respect to the frequency at room temperature is shown in Fig. 5c. The ac conductivity for all samples was calculated using Eq. (5)^22^.

5 $${\text{ac Conductivity}} = \epsilon_{{\text{r}}} \varepsilon_{{\text{o}}} {\upomega }$$

The ac conductivity is considered a function of frequency. The graph shows that the ac conductivity increases with increasing the applied frequency. Ac conductivity is small at low frequencies, which may come from charges building up at the grain boundaries; as the frequency goes towards higher values, the hopping rate of the free charge carriers and the displacement current due to the bound charges increase, thus increasing the ac conductivity. The ac conductivity also varies with the Mn concentration and reduces with increasing doping. This may be attributed to the blockage of charge carriers at the grain boundaries as the defects ions in the ZnO lattice also increase with increasing doping. Mn doping has the effect of making ZnO's grain size smaller. The formation of smaller grains can be sparked by Mn atoms, which can enhance the material's electrical characteristics. The presence of Mn can also lessen the material's flaws, which can improve the grain boundary qualities even more. These observations assisted us in understanding the influences of Mn doping in different proportions and how they affect dielectric behavior and deliver a certain amount of control over them^10^.

## Conclusion

We have successfully fabricated the Manganese Doped Zinc oxide nano-particles of various concentrations (0.0, 0.05, 0.10, 0.15, and 0.20) by the co-precipitation method. The phase purity, crystallinity, and formation of the hexagonal structure were observed from XRD analysis. We have observed that the crystallite size is greatly affected by Mn concentration. Scanning electron microscopy demonstrated well-dispersed spherical particles within the range of 40-50 nm. The Results of UV spectroscopy showed changes in the band gap by varying doping concentration. The band gap observed using the Tauc plot decreases with increasing Mn concentration due to s-p-d exchange interaction between the dopant and host. This shows we can tune ZnO optical band gap and hence it can prove Mn-doped ZnO, a favorable candidate for optoelectronics, spintronics, micro and nano-devices orbitals. The dielectric studies showed a decrease in the dielectric constant(real part), dielectric loss factor(imaginary part) and the ac conductivity with increasing Mn concentration, as introducing Mn in the ZnO structure reduces the overall polarity, which results in a decrease of the above-mentioned dielectric properties. It is concluded that the effect of different dopant concentrations achieved the tremendous goal of this research work, which leads to obtaining good optical, dielectric, and structural properties of these materials for future applications.

## Data Availability

The authors confirm that the data supporting the findings of this study are available within the article.
